# Beyond Prison Walls: A Scoping Review of Incarceration’s Public Health Impacts in Latin America

**DOI:** 10.12688/wellcomeopenres.26021.1

**Published:** 2026-03-27

**Authors:** Daniela Larrain, Caroline M. Parker

**Affiliations:** 1 Anthropology, University College London, London, WC1H 0BW, UK

**Keywords:** Incarceration, Family Health, Community Health, Latin America

## Abstract

**Background:**

Incarceration has vast and unequal impacts on public health beyond prison walls. Research from the United States documents these collateral consequences, including elevated mental illness and morbidity for the children and partners of incarcerated individuals, alongside community-level effects such as increased rates of teenage pregnancy and multidrug-resistant tuberculosis. In Latin America, however, these broader health impacts remain critically understudied. A significant barrier is the absence of data collection efforts or theoretical frameworks for mapping how Latin America’s prisons affect the health of families and surrounding communities.

**Objective:**

This scoping review synthesises existing evidence on the collateral health consequences of incarceration in Latin America by conducting a comprehensive search in 2025 that included English, Spanish and Portuguese language peer-reviewed studies.

**Results:**

From 17 included documents, prisons emerge as epicentres for tuberculosis transmission to visiting families and surrounding communities. The evidence reveals significant mental and physical health burdens on families. Women with incarcerated partners experience depression and anxiety whilst managing economic strain and expanded caregiving responsibilities, often neglecting their own health. Children of incarcerated parents show marked emotional distress, and incarcerated mothers alongside their young children face severely inadequate healthcare access within detention spaces.

**Conclusions:**

Despite collecting demographic data, most studies overlook how these burdens fall unequally across racialised populations. Future research must centre ethnoracial disparities and situated knowledge.

## Plain Language summary

Incarceration in Latin America has far-reaching effects on partners, children, families, and communities beyond prisons walls. While we know prisons’ health impacts on incarcerated people, we know far less about these broader impacts on families and communities. Most research comes from the United States, but Latin American prisons face different challenges, from severe overcrowding to limited healthcare access.

In this study, researchers searched for all available evidence on incarceration’s health consequences for people outside prison walls in Latin America, finding 17 studies published in English, Spanish, and Portuguese examining impacts on partners, children, families, and communities.

The evidence reveals that Latin American prisons act as amplifiers of infectious diseases, particularly tuberculosis, which spreads to families during visits and to communities after release. Beyond infectious diseases, the research shows troubling patterns for families, especially women. Female partners of incarcerated men experience high rates of depression and anxiety, and some studies found elevated stress hormones and increased cardiovascular disease risk among these women. Many neglect their own healthcare while managing financial strain, emotional burden, and increased caregiving responsibilities for children and family.

Children and families experience significant emotional distress, intensified during events like COVID-19 visitation bans. The review also identified inadequate prenatal and child healthcare for pregnant women and young children living with their mothers in prison due to poor detention conditions.

Understanding incarceration’s impacts on family and community health is essential for developing effective public health policies. A significant limitation appeared in how studies handled ethnoracial diversity: although some collected this information, it rarely informed health impact analysis. This oversight is particularly troubling in a region with diverse Indigenous and Afro-descendant populations, where robust evidence demonstrates pervasive racial inequalities across health, criminal justice, and other socioeconomic metrics. Future research must compile ethnoracial data informed by Latin American and locally-situated knowledge.

## Introduction

In recent years, our understanding of incarceration has shifted drastically. What was previously considered a purely “political” issue is now also recognized as a pressing public health problem ( Bowleg, 2020; LeMasters et al., 2022; Wildeman & Wang, 2017). Prisons have far-reaching collateral effects on health that extend well beyond their walls. Evidence from the United States illustrates its extensive collateral effects: Children of incarcerated parents face elevated rates of ADHD, depression, and asthma ( Lee, Fang, and Luo 2013; Wildeman, Goldman, and Turney 2018). Partners, primarily women, experience higher rates of cardiovascular issues, obesity, and sexually transmitted infections ( Khan et al. 2011; Lee et al., 2014). At the community level, high incarceration rates have been linked to lower neighbourhood life expectancy, increased teenage pregnancy and multidrug-resistant tuberculosis ( Gygli et al., 2021; Holaday et al., 2025; Thomas & Torrone, 2008). These effects are not evenly distributed. Due to staggering racial disparities, incarceration is now recognized as a significant driver of racial health inequity ( Brinkley-Rubinstein & Cloud, 2020).

Yet in Latin America—where a doubling of the prison population over twenty years has landed 1.5 million people behind bars ( World Prison Brief, 2020)—research into incarceration’s broader public health impacts is strikingly limited. While a robust literature describes health problems that occur inside Latin American prisons—notably infectious disease outbreaks of HBV, HCV, HIV, and leprosy ( Magri et al., 2025), chronic diseases like hypertension and diabetes ( Hachbardt et al., 2020; Shabil et al., 2025), and mental illnesses and substance use disorders ( Albertie et al., 2017; Gabrysch et al., 2019; González-Riera et al., 2024)—this internal focus has overlooked incarceration’s collateral health impacts beyond the prison. Notably, tuberculosis (TB) is the key exception, with evidence from Argentina, Brazil, Colombia, El Salvador, Mexico, and Peru suggesting that prison expansions since 1990 have exported TB into surrounding communities, driving Latin America’s higher-than-expected regional incidence by an estimated 29.4% ( Liu et al., 2024).

Generating robust data on the public health impacts of incarceration beyond prison walls is a crucial first step toward addressing its harms. This scoping study maps the existing research to pinpoint critical gaps and synthesize evidence. By consolidating findings on incarceration’s health effects for families and communities, it aims to catalyse future research and illuminate the full scope of incarceration’s public health burden across Latin America.

## Methods

The scarcity of empirical research on the collateral health impacts of incarceration in Latin America necessitates a methodological approach capable of mapping a fragmented literature. We therefore employed a scoping review to classify existing evidence, map the current knowledge landscape, and pinpoint critical gaps ( Peters et al., 2020).

For the scoping review, we selected key words and search terms (“incarceration” OR “imprisonment” OR “prison” OR “criminal justice” OR “corrections”) AND (“incarcerated people” OR “incarcerated individuals” OR “prisoners”) AND (“health” OR “wellbeing” OR “mental health” OR “epidemic” OR “disease” OR “illness” OR “mortality” OR “morbidity” OR “healthcare access” OR “health disparities”) AND (“Argentina” OR “Bolivia” OR “Brazil” OR “Chile” OR “Colombia” OR “Costa Rica” OR “Cuba” OR “Dominican Republic” OR “Ecuador” OR “El Salvador” OR “Guatemala” OR “Haiti” OR “Honduras” OR “Mexico” OR “Nicaragua” OR “Panama” OR “Paraguay” OR “Peru” OR “Puerto Rico” OR “Uruguay” OR “Venezuela” OR “Caribbean” OR “Latin America” OR “South America” OR “Central America”). The search was conducted across multiple academic databases, including MEDLINE (PubMed), Scopus, Web of Science and EBSCOhost, ensuring broad coverage of health and social sciences research related to incarceration in Latin America.

To capture the region’s scholarly output comprehensively, the review was conducted between September and December of 2025 and included studies published in English, Spanish, and Portuguese. Our selection prioritized methodological diversity within the peer-reviewed literature, incorporating qualitative and quantitative studies. To ensure contemporary relevance, the review included only articles published since 2000, capturing evidence from the era of Latin American prison expansion that began in the 1990s ( Parker & Weegels, 2023).

We conducted a two-stage search and screening process to identify relevant literature.

### Stage 1: Comprehensive search on incarceration’s health impacts

An initial search identified 1,532 studies on incarceration’s health impacts in Latin America (1,269 in English, 86 in Spanish, 177 in Portuguese). After importing these into Covidence (Veritas Health Innovation, Melbourne, Australia), removing duplicates yielded a corpus of 842 studies for screening. During the initial title/abstract screening, we retained all peer-reviewed qualitative or quantitative studies published from 2000 onwards that examined any health outcome—whether affecting incarcerated individuals, their families, or communities—related to incarceration in Latin American contexts. This screening excluded 344 studies, yielding 498 studies (399 English, 37 Spanish, 62 Portuguese) eligible for full-text assessment.

### Stage 2: Refinement to family and community health impacts

**Figure 1.**
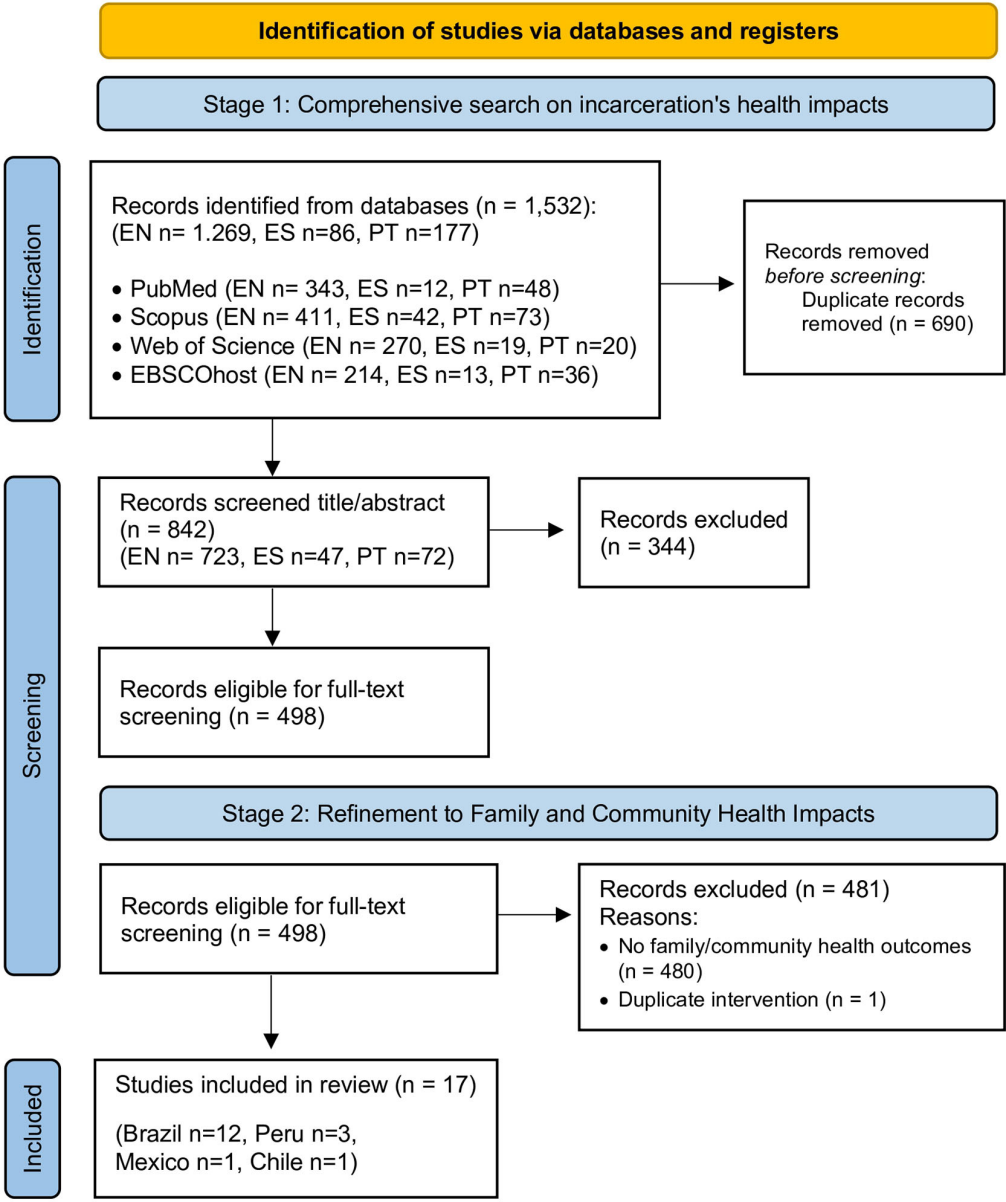
Flow diagram outlining the study search and selection process. This figure outlines the identification, screening, and inclusion of studies across two stages. A database search across PubMed, Scopus, Web of Science, and EBSCOhost yielded 1,532 records in three languages. Following duplicate removal and title/abstract screening, 498 records underwent full-text review. Stage 1 assessed incarceration’s health impacts broadly, while Stage 2 refined selection to family and community health outcomes. Seventeen studies were ultimately included (Brazil n = 12, Peru n = 3, Mexico n = 1, Chile n = 1). EN = English; ES = Spanish; PT = Portuguese; n = Number of studies.

Given the large volume of studies identified in Stage 1—many focusing exclusively on the health of incarcerated individuals—we refined this corpus by applying thematic criteria that prioritized health impacts beyond prison walls. Specifically, studies were included if they described the health of family members of incarcerated people or if they described a community-level health outcome associated with incarceration. Studies were excluded if they focused only on the health of incarcerated individuals, with one exception to this exclusion criterion: we retained studies of incarcerated mothers who either gave birth in prison or live in prison with their children, as this population represents a critical nexus where the prison environment directly shapes family and child health outcomes. Additionally, we excluded studies if they described the same data as another article. The full-text review excluded 481 studies. This yielded a final corpus of 17 studies for data extraction and synthesis (see Figure 1), with a geographic distribution that was heavily concentrated in Brazil (n = 12), with the remaining studies from Peru (n = 3), Mexico (n = 1), and Chile (n = 1).

To structure our analysis of incarceration’s public health impacts beyond the prison, the review first organizes the literature by population: first examining the health impacts on non-incarcerated partners and other family members, turning to family health inside prisons including maternal, neonatal, and child outcomes, and finally describing broader community-level health effects. Within these groupings we synthesize the evidence, outlining whether studies directly measure a health variable or use qualitative descriptions of health outcomes and behaviours, summarizing their methodologies, and noting relevant limitations. In the discussion, we reflect on gaps in the literature and identify key areas for future research. The following section summarizes the 17 studies that met our inclusion criteria, detailed in Table 1- refer to data availability statement.

## Results

### Family health beyond prison walls

Our review identified only six studies addressing the health of non-incarcerated family members ( Baccon et al., 2023; Barbosa et al., 2018; Connors et al., 2020; Martins et al., 2018; Mendes et al., 2023; Sousa et al., 2016). Two focused specifically on the female partners of incarcerated men ( Barbosa et al., 2018; Martins et al., 2018) and the rest on other female relatives or the children of incarcerated men ( Baccon et al., 2023; Connors et al., 2020; Mendes et al., 2023; Sousa et al., 2016). No studies examined the male partners or same-sex partners of incarcerated women, nor did any explore the male relatives of incarcerated women. Four of the six studies were qualitative ( Baccon et al. 2023; Martins et al. 2018; Mendes et al., 2023; Sousa et al. 2016) and did not provide a direct measure of a health variable. Notably, only one study used a control group and thus provided concrete evidence that family members of incarcerated individuals experience poor health outcomes relative their counterparts without incarcerated relatives ( Connors et al., 2020).

This study drew on the Mexican Teachers Cohort, a large cross-sectional survey of 1,849 cardiovascular disease-free female teachers ( Connors et al., 2020). Among these, 283 (15.3%) reported having an incarcerated family member. Using the Life Stressor Checklist–Revised (LSC-R) and the Perceived Stress Scale-10 (PSS-10) paired with hair cortisol analysis, the study found that women with an incarcerated family member (relation not specified) exhibited higher self-reported stress, elevated cortisol levels, and 41% higher odds of carotid atherosclerosis (95% CI = 1.04, 2.00) after multivariable adjustment. This is the only Latin American study to conclusively demonstrate a quantitative association between familial incarceration and women’s physiological stress, as measured by cortisol levels ( Connors et al., 2020).

Supporting the link between family incarceration and women’s mental health, however, comes from a cross-sectional survey of 349 female partners of incarcerated men in Paraná, Brazil ( Barbosa et al., 2018). Employing an adapted version of the Brazilian Study of Sexual Behaviour (BSSB), the survey explored chronic disease risk factors, analysing depression as the dependent variable in relation to factors including age, education, smoking, and alcohol use. A total of 42.2% of 349 female partners reported depression, with the highest prevalence among women over 30 (50.3%), smokers (61.1%), and alcohol consumers (16.1%). While the authors suggest these behaviours may serve as coping mechanisms for distress related to incarceration ( Barbosa et al., 2018), the absence of a comparison group limits conclusions about causality or baseline differences.

The remaining evidence pertaining to the health of non-incarcerated family members is entirely qualitative. A qualitative study in Paraná, Brazil, involving 19 women attending conjugal visits, suggests a potential link between male partner incarceration and heightened sexual risk behaviours ( Martins et al., 2018). All women reported engaging in unprotected sex with their incarcerated male partners during these visits. In interviews, women attributed this behaviour to trust in their partners, confidence in mandatory prison health screenings, and the use of alternative contraceptive methods. As one participant explained, “We have relationships here in the jail on the day of our visit, and I trust him a lot, because here they do all sorts of exams, and every time he does it the results are normal” ( Martins et al., 2018, p. 47). However, the study’s small sample size and lack of a comparison group for either pre-incarceration behaviours or for women whose partners are not incarcerated makes it difficult to determine whether these sexual risks behaviours are produced by the prison environment. The same qualitative study also suggests broader health effects on women’s self-care. Outside of prison visits, women reported neglecting their own healthcare, prioritizing partners’ and children’s needs, and encountering stigma in healthcare settings. As one participant stated, “I can’t take care of my health; I have the children, so I don’t have time for myself. The time I have is mostly geared for them and to come here and bring things to him” ( Martins et al., 2018, p. 46). These insights, though preliminary, point to potential behavioural health consequences of partner incarceration.

Additional qualitative evidence comes from Baccon et al. (2023), who explored how Covid-19 visitation bans affected the families of individuals incarcerated in a high-security prison in Paraná, Brazil. Through in-depth, semi-structured interviews with 41 participants—including 28 incarcerated men and 13 of their female relatives—researchers found that prohibitions on visits caused profound anxiety and emotional suffering for both groups. Family members described a state of near-constant fear for the health and safety of their loved ones, a fear rooted in the prisons’ severe shortages of food, masks, medicine, and sanitation supplies. One relative poignantly expressed this worry directly: “… if there were no conditions out there, I kept imagining in here, it was very worrying … I was out there and worried about him, if he got contaminated and he didn’t eat properly, in prison food was very precarious” ( Baccon et al., 2023, p. 7). Interviews suggested that the psychological toll may have been especially acute for children separated from their incarcerated parents. One participant noted a painful regression in her young son: “… My four-year-old son is autistic. When my husband was arrested, he called him every night (he cried), and that went away, and he forgot about his father” ( Baccon et al., 2023, p. 8). Beyond the emotional strain placed on relatives, bans were also said to have imposed significant economic hardship on families who were compelled to send essential items like food, masks, and medicine via courier to compensate for the prison’s inadequate provisions, creating an additional financial burden during the Covid-19 epidemic.

Although not a study of family health per se, qualitative research with incarcerated men describes how these men perceive their imprisonment to impact their families’ wellbeing. One such study, based on interviews with ten men on probation in Mato Grosso do Sul, Brazil, found that families—and particularly mothers—often became primary sources of emotional and material support amid institutional neglect ( Mendes et al., 2023). One participant highlighted this reliance, stating: “My mom is helping me a lot, she doesn’t throw it in my face, she only brings good things. She used to visit me in prison, she still visits me, she gives me support. After I get away from here I’ll go to her house. My brother and my father never visited” ( Mendes et al., 2023, p. 5). Interviews frequently highlighted the prison system’s failure to provide timely medical care. This institutional neglect forced incarcerated men to rely on their own families or, at times, the families of fellow cell-mates to meet their healthcare needs. As one participant reported: “I was working with a chemical, it hit my eye, it burned. I felt a lot of pain in the maximum security. I could only get medicine months later and because of the inmates, their families …” ( Mendes et al., 2023, p. 5).

Further qualitative evidence for incarceration’s impact on families comes from interviews with twenty men facing charges related to conjugal violence in Salvador, Brazil ( Sousa et al., 2016). In interviews, men described the impact of their imprisonment on their families, citing forced separation from their children due to restraining orders, severe economic strain from lost employment, and profound disruption to family relationships, particularly for their children. One participant’s statement illustrates this last consequence: “My children are living with serious problems (silence, eyes filled with tears). The boy is not going to school, the girl is struggling to study. I see that if we do not keep our children, they will find a way to throw themselves in this world” ( Sousa et al., 2016, p. 3). Notably, the men’s accounts centred on the crisis of their absence, emphasizing their perceived inability to provide economically and guide their children, while largely not addressing how their prior domestic violence impacted their families’ wellbeing. Nevertheless, this study suggests potential pathways through which domestic violence and paternal incarceration may influence childhood, even if it cannot assess the relative wellbeing of children with an incarcerated father versus those living in a household with a father perpetrating domestic violence.

### Family health within prison

Our review identified seven studies addressing family health within prisons, a literature that is exclusively maternal, focusing on the health and wellbeing of incarcerated mothers co-residing in prison with their children, including those born in custody ( Cavalcanti et al., 2018; Domingues et al., 2017; Ferreira et al., 2021; Martínez-Álvarez & Sindeev, 2021; D. S. S. dos Santos et al., 2025a; M. V. dos Santos et al., 2025b; Vildoso-Cabrera et al., 2024). The most methodologically rigorous study we found was a nationwide study in Brazil by Domingues et al. (2017), which compared the health outcomes of incarcerated mothers and their babies born in custody against those of the general population. This study used two datasets: the “Birth in Brazil” survey (n = 16,931 non-incarcerated women, excluding minors and privately-funded births) and the “Maternal and Infant Health in Prisons” study (n = 241 incarcerated mothers from 33 prisons). Its comparative design clearly documented that systemic inadequacies in prenatal care for incarcerated women directly led to significantly worse health outcomes. The data revealed substantial deficits across all antenatal care indicators for the incarcerated cohort. For instance, only 48.1% initiated care early (vs. 60% non-incarcerated), and just 48% completed an adequate number of consultations (vs. 73%). Crucially, testing rates for infectious diseases were markedly lower, with only 68.2% of incarcerated women receiving a syphilis test (vs. 88.3%) and 69.2% an HIV test (vs. 80%). These care disparities translated into severe clinical outcomes. The study demonstrated a 12.6-fold higher incidence of congenital syphilis in children born in prison. The mother-to-child transmission rate of syphilis was nearly double among incarcerated women (66.7% vs. 36.8%). Furthermore, the prevalence of syphilis and HIV during pregnancy was significantly higher in the incarcerated group (8.7% and 3.3%, respectively) than in the general population (1.3% and 0.5%), underscoring the compounded health impact of incarceration.

The remaining six studies ( Cavalcanti et al., 2018; Ferreira et al., 2021; Martínez-Álvarez & Sindeev, 2021; D. S. S. dos Santos et al., 2025a; M. V. dos Santos et al., 2025b; Vildoso-Cabrera et al., 2024) provide valuable descriptive insights into the wellbeing of mothers and children living in prison but are limited by their lack of control groups and concrete health measures, which restricts causal inference. For example, a qualitative study of thirteen mothers incarcerated with their young infants (aged 2–27 months) in Lima’s Women’s Chorrillos Penitentiary captured mothers’ significant distress related to childbirth in prison and its perceived impact on child wellbeing ( Martínez-Álvarez & Sindeev, 2021). In interviews, a recurring theme was mothers’ associated guilt, anxiety, and worry over their ability to provide care. One mother’s account poignantly illustrated the anguish of separation from her older children: “… I feel really bad because I call my children and they ask me why they can’t see me, why I don’t take them to school, why I’m not helping them with their homework [crying]” ( Martínez-Álvarez & Sindeev, 2021, p. 102). Impending, mandatory separation from co-residing children at age three was a particularly traumatic prospect; one mother said leaving her child would be “the most painful day of her life” ( Martínez-Álvarez & Sindeev, 2021, p. 102). All participants gave birth in public hospitals while handcuffed, where they reported discrimination and restrictive security. One recounted: “… I felt bad, I couldn’t rest, I couldn’t sit down because I was handcuffed too, chained to the bed when I gave birth … really horrible …” ( Martínez-Álvarez & Sindeev, 2021, p. 102).

In the same study, mothers described multiple perceived negative impacts of the prison environment on their children’s health, particularly concerning nutritional and dental outcomes. They reported that inadequate prison food quality and quantity contributed to what they perceived as nutritional deficiencies in their children, including symptoms they associated with anaemia and low haemoglobin levels. Children were also described as experiencing dental problems attributed to the prison environment. One mother’s account synthesized these concerns: “… the babies break, break the door, they kick and scream, my boy has colic, I can’t get out … the food for the children? … all the babies here have low haemoglobin and their teeth are bad …” ( Martínez-Álvarez & Sindeev, 2021, p. 103). Mothers further linked cold, damp, and unsanitary living conditions—along with exposure to second-hand smoke—to their children’s recurrent respiratory symptoms and illnesses diagnosed as lung disease. One participant reported: “… then my daughter ended up with lung disease because I live in damp conditions, it’s all made of cement, the place where I am is very cold for her” ( Martínez-Álvarez & Sindeev, 2021, p. 103). Access to paediatric care was another critical issue: participants reported that general practitioners refused to treat their children, stating they were not paediatric specialists and lacked expertise in childhood conditions, thereby compromising the children’s healthcare.

Expanding on this understanding of maternal experience, a related qualitative study at a Prison Unit in Ceará, Brazil, used story-drawing techniques with 17 participants (including pregnant women and mothers with children in the nursery) to explore caregiving in prison ( Ferreira et al., 2021). Participants created drawings representing childcare, which were then analysed individually with each mother before researchers thematically categorized the content. The analysis of drawings and maternal narratives identified several key impacts of incarceration on health and caregiving. All participants reported significant sleep impairment, prioritizing their children’s comfort in overcrowded cells with shared beds. Despite severe constraints, mothers maintained essential care practices like breastfeeding, bathing, and food preparation. However, systemic challenges were substantial; for example, weekly food provisions for children were consistently inadequate. Researchers also noted gaps in maternal childcare knowledge, such as misconceptions about umbilical stump care. A key finding from the visual data was that motherhood was portrayed as transcending the prison walls. Mothers depicted all their children—both those co-residing and those outside the facility—with equal proximity and detail, visually reflecting their reported ongoing emotional bonds and preoccupying worry regarding separated children.

Parallel ethnographic research from Brazil sheds further light on how the prison environment itself harms child development ( D. S. S. dos Santos et al., 2025a). Through semi-structured interviews and observation with incarcerated mothers, health professionals, and officers across two units, the study exposed the profound lack of recreational or stimulating environments for children, severely limiting their opportunities for social interaction. Health professionals provided vivid testimony: one described rampant skin ailments, noting, “it is very hot in the cell and diapers are hardly made available by the administration, when the family and the Prison Ministry do not provide them, it is very complicated, then the children end up with diaper rash. All children had some kind of skin problem, such as prickly heat, itching and diaper rash” ( D. S. S. dos Santos et al., 2025a, p. 4). Another emphasized profound psychological damage, stating it is “impossible to get out of this hell without any consequences” as children are “deprived of social contact and interaction with other children” ( D. S. S. dos Santos et al., 2025a, p. 4).

Prison health records provides another valuable source of evidence for how incarceration impacts the health of children born in prison. An analysis of prison records from the Socabaya-Arequipa-Peru Women’s Prison explored incident logs, medical reports, and activity control books pertaining to 11 incarcerated mothers and the 8 children residing with them, within a total inmate population of 184 ( Vildoso-Cabrera et al., 2024). It found that children experienced frequent illness and inadequate conditions, leading some mothers to opt for early separation. Specifically, four mothers elected for early separation due to their children’s recurrent illnesses. A further two actively considered ending cohabitation, believing their children would have better living standards outside and citing responsibilities to other offspring. Visitation patterns were notably irregular, a situation exacerbated by the facility’s inadequate reception and recreational spaces—as a converted military fort lacking architectural adaptation for children. Moreover, the logs recorded persistent diet- and hygiene-related illnesses among children.

Two studies focused specifically on breastfeeding in prison ( Cavalcanti et al., 2018; M. V. dos Santos et al., 2025b). One was a qualitative study of seven lactating women in a Brazilian maternal-child prison unit, which examined how institutional environment shapes the practice of breastfeeding ( M. V. dos Santos et al., 2025b). Strikingly, the incarcerated mothers described the unit in protective terms, crediting its essential structure for child survival; as one stated, “Without this structure, many children would not be alive” ( M. V. dos Santos et al., 2025b, p. 5). Yet, they simultaneously described the profound loss of external family support as a primary source of strain. As another mother explained, “I need help, I’m afraid” ( M. V. dos Santos et al., 2025b, p. 6), directly linking her isolation to a crisis in maternal confidence. This dual finding delineates some of the specific ways that prisons and motherhood are entangled: while maternal units offer essential support to incarcerated mothers, they simultaneously deepen their isolation, precisely because they remain, after all, prisons.

The only other study we identified to explore a health-relevant variable of motherhood behind bars was a cross-sectional quantitative study conducted in four Brazilian prisons, which examined breastfeeding practices among incarcerated mothers residing with their infants (1–5 months old) ( Cavalcanti et al., 2018). This analysis, based on a sample of 13 incarcerated mothers, found that although most participants received prenatal care (60%) and breastfeeding education regarding WHO guidelines (90.9%), only 33.3% maintained exclusive breastfeeding for two months, falling short of the WHO’s six-month guideline. In fact, all infants were bottle-fed within three months and all used pacifiers from birth. The authors concluded that non-adherence to this guideline was linked to insufficient knowledge, poor institutional promotion, and the compounded stressors of incarceration.

### Community health in the shadow of the prison

Just four studies documented incarceration’s impacts on community level health, all focusing exclusively on tuberculosis (TB) transmission ( Aguilera et al., 2016; Mabud et al., 2019; Sacchi et al., 2015; Warren et al., 2018). The most extensive was a population-based study in Dourados, Brazil, which revealed prisons act as key reservoirs for community TB spread ( Sacchi et al., 2015): 54% of community strains were genetically linked to prison strains. Incarcerated individuals had TB rates 40 times higher than the general population (1,044 vs. 26 per 100,000), and a case-control analysis identified prior incarceration as the strongest independent risk factor for community TB, accounting for 23% of cases. Notably, 83% of formerly incarcerated people with TB were diagnosed within two years of release, suggesting infection occurred during incarceration. Molecular typing of bacterial isolates further confirmed prisons as a substantial source of community transmission, indicating that inadequately controlled transmission within prisons substantially elevates TB burden in surrounding populations.

An observational and modelling study in Brazil further demonstrated that tuberculosis transmission within prisons produces substantial and prolonged spillover into the wider community ( Mabud et al., 2019). By linking national TB registry data with state prison records from 2007–2013, researchers identified 615 TB cases among 42,925 incarcerated and formerly incarcerated individuals. During incarceration, TB incidence peaked at 1,303 per 100,000 person-years—30 times higher than the general population. Critically, this elevated risk persisted after release, with an incidence rate of 229 per 100,000 person-years (5.5 times the community baseline) remaining for seven years before declining. Each additional year of incarceration increased post-release TB risk by 32% (aHR 1.32, 95% CI 1.19–1.48), confirming that prison exposure directly elevates community TB burden through released individuals.

Similarly, a cross-sectional study by Aguilera et al. (2016) across 46 Chilean prisons establishes that carceral facilities function as powerful accelerators of tuberculosis (TB) transmission, with direct spillover into the community. The research quantified a stark, 13-fold higher TB incidence among incarcerated individuals compared to the general Chilean population. The study’s cohort of 418 contacts—including 328 prisoners, 41 healthcare workers, 29 guards, and 20 community visitors—revealed a distinct infection gradient. Prisoners bore the highest latent infection (LTBI) burden at 33.2%, while the 25% LTBI prevalence documented among community visitors provides concrete evidence of transmission pathways that bridge prison walls.

Finally, a spatial and molecular study in Lima, Peru, tracked the spread of multidrug-resistant tuberculosis (MDR-TB) from a prison to surrounding neighbourhoods ( Warren et al., 2018). Modelling data from 1,587 tuberculosis patients (164 with MDR-TB), researchers established a 5.47-kilometer spillover zone around the prison, within which the MDR-TB prevalence was significantly higher (14.8% vs. 8.2%). Genetic analysis confirmed this spatial link: strains from community patients within the zone were more than twice as likely to match those of current inmates. Furthermore, four of eight community clusters of genetically identical MDR-TB cases were located within the prison’s spillover area, indicating that the facility served as an ongoing source seeding local transmission networks.

## Discussion

This scoping review identifies a critical and geographically narrow evidence base regarding the public health impacts of incarceration beyond prison walls in Latin America. Research in this domain is not only scarce but overwhelmingly concentrated in Brazil, with only a handful of studies emerging from the region’s numerous other nations. The existing literature is predominantly qualitative, and the limited quantitative studies that directly measure health outcomes are almost exclusively observational and lack control groups. This compounded deficit—both in geographic scope and methodological rigour—severely constrains causal inference and underscores a profound gap in Latin American public health research.

Nevertheless, by synthesizing the available evidence, a hierarchy of findings emerges, ranging from demonstrated causal pathways to plausible but unproven associations. The most robust evidence points to a direct causal mechanism: prisons function as powerful institutional amplifiers of infectious disease, which then spill over into surrounding communities. In Latin America, this is most conclusively established for tuberculosis (TB), where a series of rigorous epidemiological and molecular studies across Brazil, Chile, and Peru provide definitive proof. These studies document prison TB incidence rates that are 13 to 40 times higher than general population rates and employ genetic sequencing to directly link community TB and multidrug-resistant TB (MDR-TB) cases to prison reservoirs ( Aguilera et al., 2016; Sacchi et al., 2015; Warren et al., 2018).

While TB is the primary documented example in this region, evidence from the United States demonstrates that incarceration is a significant driver of community transmission for other infectious diseases, including HIV and COVID-19 ( Johnson & Raphael, 2009; Krebs & Simmons, 2002; Reinhart & Chen, 2021). This indicates a critical gap in the Latin American literature and underscores the need for parallel research to determine whether similar spillover dynamics exist for other concentrated infections.

In the Latin American context, beyond the clear case of TB, the evidence shifts from causation to association. A smaller body of controlled, quantitative research identifies significant links between familial incarceration and adverse health markers, though causality remains ambiguous. The strongest of these associations link familial incarceration to heightened stress, elevated cortisol, and increased odds of carotid atherosclerosis in female relatives ( Connors et al., 2020). A separate, compelling line of comparative evidence shows that systemic failures in prenatal care for incarcerated women are associated with starkly worse outcomes, including a 12.6-fold higher incidence of congenital syphilis in their newborns compared to the general population ( Domingues et al., 2017). These studies provide the most robust quantitative evidence of harm identified in this review, yet they cannot definitively isolate the effect of incarceration from the profound, pre-existing vulnerabilities of the affected populations.

Finally, a larger corpus of qualitative and uncontrolled observational research suggests a wider spectrum of potential health impacts, though these remain hypotheses for future validation. This literature points to significant mental health burdens, including high rates of depression among female partners of incarcerated men ( Barbosa et al., 2018) and profound anxiety and emotional suffering in families, particularly during disruptive events like Covid-19 visitation bans ( Baccon et al., 2023). It also suggests sexual risks behaviours including condomless sex during prison visits ( Martins et al., 2018), the neglect of personal healthcare by women managing the consequences of a partner’s incarceration, and the intergenerational strain placed on families—often mothers—who become primary caregivers for incarcerated relatives ( Mendes et al., 2023; Sousa et al., 2016). Within prison walls, qualitative studies describe perceived physical and developmental harms to co-residing children and the intense psychological strain on incarcerated mothers. These accounts depict a paradoxical system: while providing some essential maternal support, it simultaneously subjects women to degrading conditions—including giving birth while chained to beds—and exposes both mothers and children to profoundly hostile prison environments ( Martínez-Álvarez & Sindeev, 2021; M. V. dos Santos et al., 2025b).

This hierarchy—from proven causal transmission to suggested behavioural pathways—reveals two concurrent dynamics. The first is an established epidemiological fact: incarceration demonstrably creates and exports novel population-level health threats, as clearly evidenced by TB. The second is a critical hypothesis: incarceration concentrates individuals and families with profound, often intergenerational, vulnerabilities into an environment that fails to address—and may actively intensify—their pre-existing health crises. Disentangling these concurrent realities is the central methodological challenge. Causal attribution is deeply complicated by the substantial pre-existing vulnerabilities that characterize the population entering the carceral system. Quantitative profiles of incarcerated women in Brazil and Peru, for example, illustrate a distinct pre-incarceration health profile marked by significant childhood adversity, including abuse linked to intergenerational cycles of violence ( Falbo et al., 2004), a history of sex work and STIs ( Nicolau et al., 2012), and substance use disorders nearly tenfold higher than general population estimates ( Cyrus et al., 2021). These compounded vulnerabilities intrinsically affect the health of their families and social networks long before an arrest occurs. Consequently, the associations identified in this review—between familial incarceration and stress, mental health conditions, or sexual risk behaviours—exist within this context of profound baseline risk. Establishing whether incarceration itself exerts an independent causal effect on family health, rather than merely acting as a marker or intensifier of these pre-existing conditions, requires research designs capable of isolating the imprisonment event. Future studies must employ longitudinal cohorts with appropriate control groups, measure pre-incarceration health status, and analyse the specific mechanisms through which incarceration may alter health trajectories beyond the powerful influence of entrenched social deprivation.

Finally, the reviewed literature demonstrates a striking insensitivity to ethnoracial diversity. While several studies record the race or ethnicity of participants, the Mexican Teachers Cohort study ( Connors et al., 2020) study is the sole example to analyse race/ethnicity as a variable (e.g., showing family incarceration is more common among Indigenous women than non-indigenous women). Given the growing evidence base for ethnoracial disparities in Latin American incarceration ( Parker & Perez-Brumer, 2024), robust descriptive data on this distribution is a critical prerequisite for understanding its public health impacts. Future research must, therefore, systematically collect and analyse ethnoracial data—ideally grounded in Latin American epistemologies and local understandings of ethnoracial diversity.

As well as researchers, government and policy play a critical role. Across Latin America, incarceration is almost never included as a variable in routine health and social surveys, rendering the health consequences of the world’s largest prison boom this century profoundly difficult to measure. To make these impacts visible, national governments and public health bodies must systematically integrate incarceration variables into data collection. Platforms like the Demographic and Health Surveys ( DHS, 2025) and Multiple Indicator Cluster Surveys ( UNICEF, 2025) could be readily adapted to include questions about parental incarceration. Nationally, school-based surveys are vital for tracing incarceration’s impacts on children. Only through such rigorous, sustained data collection can the true scale of incarceration’s public health burden be concretely established and addressed.

This scoping review has certain limitations. As a scoping review rather than a systematic review, this study does not encompass an exhaustive bibliography of all published academic articles on the topic. Furthermore, it has not appraised the quality of evidence of included studies or evaluated intervention effectiveness.

This table summarises health outcomes associated with incarceration across three domains: family health outside prison, family health inside prison, and community health. Outcomes are organised by health category (mental health and behavioural, chronic and non-communicable, and infectious and communicable) and characterised by strength of evidence and study design. n = number of studies.

## Data Availability

Zenodo: Extended data_Table 1.docx. Doi: https://doi.org/10.5281/zenodo.18564752 ( Larraín, D., & Parker, C. (2026)). Licence: Creative Commons Zero v1.0 Universal. PRISMA-ScR Checklist: The completed PRISMA Extension for Scoping Reviews (PRISMA-ScR) checklist for this study is publicly available in Zenodo: Larraín, D., & Parker, C. (2026). PRISMA-ScR Checklist for Beyond Prison Walls: A Scoping Review of Incarceration’s Public Health Impacts in Latin America. Zenodo. https://doi.org/10.5281/zenodo.18564752. Licence: Creative Commons Zero v1.0 Universal. This review synthesises existing published literature and does not involve collection of new data. All studies included in the review are referenced and can be accessed through their original publishers. The extracted data and characteristics of included studies appear in the manuscript and Table 1.
