# Supplementary material for: Beyond Prison Walls: A Scoping Review of Incarceration’s Public Health Impacts in Latin America
Source: Wellcome Open Res. 2026 Mar 27;11:193. [Version 1] doi: 10.12688/wellcomeopenres.26021.1 (PMC13367167; doi:10.12688/wellcomeopenres.26021.1)
Supplement: Peer review history

# Reviewer report: Reviewer response for version 1

**DOI:** 10.21956/wellcomeopenres.28646.r156186

**Published:** 2026-06-13

**Authors:** Nicholas Iacobelli

**Affiliations:** 1 University of Washington, Seattle, Washington, USA

The authors of this scoping review present the available evidence for the wide-ranging health effects of incarceration that extend outside carceral facilities in Latin America to affect communities and family members. As they acknowledge, the data are regrettably sparse. However, with that data they are able to extract and present meaningful conclusions about those effects and importantly highlight a path forward for future investigation.

 In the introduction, the authors helpfully contrast availability of data in Latin America with that of the United States (US), though the sentence that outlines lower life expectancy, higher rates of teenage pregnancy, and higher rates of multidrug resistant tuberculosis (MDR-TB) is a little confusing in this context. It seems to flow from commentary about data in the US and is cited with other studies about the US. However, there is little evidence of higher rates of community level MDR-TB in the US associated with incarceration due to lower burden of disease especially of MDR strains (though rates of TB among incarcerated people are still higher than the general public), longer incarceration times, and improved testing and treatment. The reference for this claim is from the country of Georgia, so I would recommend rephrasing these sentences to be clearer and more specific. A recent scoping review of community health effects in the US offers a helpful comparison to this review. ^1^ The authors in that review cite the lack of association with higher TB community TB rates and also references a study demonstrating higher community STI rates, ^2^ which demonstrates at least one way infectious disease rates, even if not TB, are higher in the community as a consequence of incarceration.

 The introduction also references a doubling of the prison population in Latin America over the last 20 years. While the incarceration rates in the US are still much higher, the rate of increase in the last 20 is lower. Although outside the scope of this review, it would be helpful to provide some context about what the drivers of this surge in incarceration rates are in Latin America and if it is heterogeneously experienced across Latin American nations (e.g. with the highest burden of the surge in Brazil, El Salvador, and Ecuador). This last point would also help lend context to the representativeness of these nations in the study data.

 The methodology for the review is sound, with appropriate search terms and language inclusion for the compiling of relevant studies. It is notable as the authors point out that most of the studies were from Brazil and were qualitative. Although the authors mention the breakdown of qualitative and quantitative studies in the text, it would also be helpful to represent this in the chart in Figure 1. For reasons I will outline later, while the inclusion criteria of “described health of family members of incarcerated people” allowed for compiling relevant qualitative and quantitative data, it may have led to the inclusion of at least one study that is unable to relate health characteristics to incarceration in a meaningful way.

 The authors did a good job of organizing the very heterogeneous data to present important conclusions and highlighting the results, especially as they pertain to the review’s aims. In particular, they were able to extract relevant quotations from qualitative studies that not only capture the study’s findings but relate back to the aims and purpose of the review. The authors frequently discuss lack of control or comparison group for qualitative studies, which I do not think is a particularly relevant metric. A more meaningful parameter for qualitative studies would be to assess either how the authors of those studies frame the connection between the health/social effects described and incarceration or how the participants themselves framed it in their narratives as presented. The mix of qualitative and quantitative studies, though, does reinforce the strength of some of these findings and allow for more causal relationships to be inferred.

 With respect to Barbosa et al., 2018, I agree with its inclusion based on the criteria presented in the methods section. However, it seems to use female partners of incarcerated men as its primary sample group with depression as a dependent variable and age, education, smoking, and alcohol use as independent variables, drawing a correlation between these factors and higher rates of depression. Unfortunately, without even a reference to rates of depression in the general population, it is unable to provide any meaningful commentary about depression as an impact of incarceration, which is the purpose of this review. For what it is worth, it does cite lower rates of smoking in the general population in Brazil, so one can infer based on the associations they show that rates of depression would also be higher among women with incarcerated partners. Given this data, although the authors state they do not appraise the quality of evidence, I would recommend that they be more explicit about this study’s limitations with respect to the theme of the review. For context, the authors do an excellent job of highlighting the limitations of Sousa et al, 2016 as they relate to the confounders of domestic violence.

 The authors' suggestion to pay more attention to ethnoracial diversity in the discussion is a good one, especially since studies collected the data but do not mention/analyze it, even if to say that meaningful conclusions cannot be drawn. I suggest that the authors give a bit more information about what focusing on this demographic characteristic might reveal as opposed to others. They state that there are acknowledged ethnoracial health disparities and that investigating these in the context of incarceration’s effects would be informative, though presumably the same is true for other demographic variables (e.g., gender, class). This recommendation seems slightly out of place with what I found to be the much stronger and more impressive recommendation to gather more data with specifics on how—and the ease with which—this could be done when combined with routine health and social surveys.

 The beginning paragraph on page 11 discusses the epidemiological fact of population-level health threats and the challenge of demonstrating causality amongst other social and structural vulnerabilities that generate confounders. I found this section of the paragraph confusing both in content and phrasing. While demonstrating causality is important, that is the strength of quantitative research. Social worlds, however, are inherently messy and less prone to being disentangled. The description of and reckoning with that complexity is the strength of qualitative research. In what is otherwise a thoughtful and well-organized review, I found that this point subjected the data to the tyranny of causality unnecessarily, when it could have focused on the relative strengths and weaknesses of the studies or what each study lends toward understanding the phenomenon of community-level health effects of incarceration.

 The discussion does a wonderful job of reviewing the context of the included studies and highlighting one of the most striking details of this review, which is the relative lack of data exploring the consequences of such a drastic surge in incarceration in the last two decades in Latin America. As the authors point out in the introduction, incarceration is a largely political (and, I would add, sociocultural) phenomenon. As much as this is the case, the framing of incarceration as a public health problem—especially insofar as it relates to causing health consequences for non-incarcerated people—is just as political. Though it may rely on problematic notions where incarceration harms the health of undeserving citizens, it rightly demonstrates that incarceration is something that affects the health of everyone and draws attention to the need for improved and equitable healthcare across the board. One might also then ask (and I encourage the authors to do so to the extent they are able in the discussion) what social and political drivers have resulted in the representation of mostly qualitative, mostly Brazilian, mostly maternal-child, mostly TB related studies.

 On the whole, I commend and thank the authors for taking on this project and providing such a robust review.

Are the rationale for, and objectives of, the Systematic Review clearly stated?

Yes

Is the statistical analysis and its interpretation appropriate?

Yes

If this is a Living Systematic Review, is the ‘living’ method appropriate and is the search schedule clearly defined and justified? (‘Living Systematic Review’ or a variation of this term should be included in the title.)

Not applicable

Are sufficient details of the methods and analysis provided to allow replication by others?

Yes

Are the conclusions drawn adequately supported by the results presented in the review?

Yes

Reviewer Expertise:

Qualitative and Ethnographic Methods; Incarceration, Health, and Healthcare; Hepatitis C; HIV; Substance Use Disorder

I confirm that I have read this submission and believe that I have an appropriate level of expertise to confirm that it is of an acceptable scientific standard.

## References

[1] : Mass incarceration and its spillover effects: a scoping review of incarceration rates and health. *Social Science & Medicine* .2026;394: 10.1016/j.socscimed.2026.118964 10.1016/j.socscimed.2026.118964 PMC1305913541666809

[2] : The contingent effect of incarceration on state health outcomes. *SSM - Population Health* .2023;21: 10.1016/j.ssmph.2022.101322 10.1016/j.ssmph.2022.101322 PMC982705236632050

# Reviewer report: Reviewer response for version 1

**DOI:** 10.21956/wellcomeopenres.28646.r155612

**Published:** 2026-06-04

**Authors:** Priscila de Morais Sato

**Affiliations:** 1 Federal University of Bahia, Bahia, Brazil

This review addresses an important and underexplored public health issue by examining the health consequences of incarceration beyond the prison walls. The aim of the review is highly relevant and timely. One of the major strengths of the manuscript is its ability to construct a compelling narrative synthesis of the included studies, clearly explaining the health problems associated with incarceration and the mechanisms through which these impacts occur. The review effectively demonstrates that the consequences of incarceration extend far beyond incarcerated individuals themselves, affecting families and communities, particularly women and children who are often already in situations of greater social vulnerability.

 The perspective of understanding the effects of incarceration “beyond walls” sheds light on an important gendered dimension of incarceration and public health. In particular, the manuscript successfully highlights how women disproportionately experience the indirect health and social burdens associated with incarceration. I also appreciated the authors' decision to retain studies of incarcerated mothers who either gave birth in prison or lived in prison with their children, as this population represents a critical nexus where the prison environment directly shapes maternal, family, and child health outcomes. However, despite these findings, gender does not appear to be explicitly discussed in the manuscript’s discussion section. Given how strongly the reviewed evidence points to gendered caregiving responsibilities and unequal social burdens, I believe a more explicit discussion of gender would strengthen the interpretation of the findings and help situate the results within broader debates on incarceration, caregiving, and structural vulnerability. In this context, it may also be valuable to reflect on the relative absence of studies examining heterosexual male partners and fathers. While this gap may reflect broader social realities in which women disproportionately assume caregiving responsibilities, it may also reproduce the invisibility of men’s social and caregiving roles within families affected by incarceration.

 I would also suggest further clarification regarding the timeline described in the methods section. The manuscript states: “To capture the region’s scholarly output comprehensively, the review was conducted between September and December of 2025.” It was not entirely clear why the search process extended across several months, as systematic and scoping review searches are often conducted within a more limited timeframe to ensure consistency and comparability in study identification. The authors could clarify whether searches were updated iteratively during this period or whether different databases were searched at different times, and how this approach may have influenced study inclusion.

 Another point that would benefit from clarification concerns the discussion of methodological rigor. The manuscript states that several qualitative studies were limited by their “lack of control groups and concrete health measures, which restricts causal inference.” While I understand the importance of causal inference for certain research questions, the current wording may unintentionally suggest that qualitative studies are inherently less rigorous because they do not employ quantitative designs or control groups. I encourage the authors to clarify the criteria used to assess methodological rigor across different study designs. Qualitative studies are often designed to provide depth, contextual understanding, and insight into lived experiences rather than causal estimation, and therefore should ideally be evaluated according to criteria appropriate to qualitative methodologies. Reframing this discussion could help avoid applying quantitative standards to qualitative work and would strengthen the methodological balance of the review.

 Overall, I found the review relevant and thoughtfully conducted. The manuscript is particularly strong in showing that the health consequences of incarceration are not restricted to incarcerated individuals, but also affect families, especially women and children. I also appreciated the focus on Latin America, where incarceration rates have increased substantially in recent decades but where the broader health and social consequences of incarceration remain comparatively underexplored in the literature. By bringing together studies from different countries in the region, the review helps make visible a dimension of incarceration that is still insufficiently discussed in public health research.

Are the rationale for, and objectives of, the Systematic Review clearly stated?

Yes

Is the statistical analysis and its interpretation appropriate?

Not applicable

If this is a Living Systematic Review, is the ‘living’ method appropriate and is the search schedule clearly defined and justified? (‘Living Systematic Review’ or a variation of this term should be included in the title.)

Not applicable

Are sufficient details of the methods and analysis provided to allow replication by others?

Partly

Are the conclusions drawn adequately supported by the results presented in the review?

Yes

Reviewer Expertise:

Public health, Gender, Race, Food insecurity

I confirm that I have read this submission and believe that I have an appropriate level of expertise to confirm that it is of an acceptable scientific standard.

# Reviewer report: Reviewer response for version 1

**DOI:** 10.21956/wellcomeopenres.28646.r152170

**Published:** 2026-04-16

**Authors:** Heather Wurtz

**Affiliations:** 1 University of Southern California, Los Angeles, California, USA

This scoping review maps the existing literature on family- and community-level impacts of incarceration in Latin America. The objectives of the scoping review are to chart current knowledge on the topic and identify gaps to guide future research.

 This is an important and underexplored area of research. The scoping review sheds light on areas in need of further exploration for future studies to improve public health knowledge and practice.

 Methods:

- When describing the final corpus of studies included in the scoping review, it might be helpful to include the overall breakdown of qual, quant, and mixed methods studies (rather than just the breakdown within each population).
- How is Latin America defined? There is not one universal system of categorization, so it might be a good idea to include a citation for how you are defining it (e.g., WHO, World Bank).

Results:

- Regarding the following statement: “Notably, only one study used a control group and thus provided concrete evidence that family members of incarcerated individuals experience poor health outcomes relative their counterparts without incarcerated relatives.” The sentence is missing “to” following “relative.”
- In the next paragraph, I would not recommend beginning a paragraph with “this study.” Perhaps replace “this” with a more specific referent.
- In the following paragraph, beginning “supporting the link…” – this sentence is hard to follow. I would reverse the order of the sentence, beginning with “a cross-sectional survey…”
- Below, regarding the following statement, “However, the study’s small sample size and lack of a comparison group for either pre-incarceration behaviours or for women whose partners are not incarcerated makes it difficult to determine whether these sexual risks behaviours are produced by the prison environment.” This sentence reads as though the small sample size and lack of comparison group were limitations to this study. But this is not the case because comparison, generalizability, causal inference, etc. are not objectives of qualitative research. I would suggest removing this sentence and instead focusing on what this study contributes to our understanding of family-level impacts of incarceration.
- Also, regarding the same paragraph, why are these insights “preliminary”? Weren’t they based on findings from this qualitative study? Would it make more sense to say something about how these insights point to hypotheses that could be further tested in future studies?

Throughout the results, there is frequent mention of the limitations of studies due to the lack of a control group. This is highlighted in the discussion, which seems fitting. I’m not sure it’s as helpful to emphasize this in the results section (especially for qualitative studies); I would suggest focusing on what the featured studies contribute to the current landscape of knowledge and then – as you do – highlighting gaps and limitations in the discussion.

 Discussion:

- In the paragraph about ethnoracial diversity, could you expand a tad on the implications of expanding knowledge of ethnoracial data for public health research and practice? How could a better understanding of ethnoracial diversity inform interventions for improving health equity and outcomes?
- Regarding the following statement, “As well as researchers, government and policy play a critical role.” A critical role in *what*?

Latin America is an incredibly diverse region in terms of culture, politics, geography, etc. I understand that the literature is limited and that grouping all Latin American countries together makes sense for the purpose of this review. But, I think somewhere this needs to be acknowledged, as well as the importance of contextualizing studies (and recommendations for interventions or policy change) within distinct historical, political, and social climates.

Are the rationale for, and objectives of, the Systematic Review clearly stated?

Yes

Is the statistical analysis and its interpretation appropriate?

Yes

If this is a Living Systematic Review, is the ‘living’ method appropriate and is the search schedule clearly defined and justified? (‘Living Systematic Review’ or a variation of this term should be included in the title.)

Not applicable

Are sufficient details of the methods and analysis provided to allow replication by others?

Yes

Are the conclusions drawn adequately supported by the results presented in the review?

Yes

Reviewer Expertise:

Qualitative and mixed methods research on health equity, which a focus on Latin American and family health outcomes.

I confirm that I have read this submission and believe that I have an appropriate level of expertise to confirm that it is of an acceptable scientific standard.
